# Right ventricle-specific therapies in acute respiratory distress syndrome: a scoping review

**DOI:** 10.1186/s13054-023-04395-9

**Published:** 2023-03-12

**Authors:** Simran Ganeriwal, Gabriele Alves dos Anjos, Mary Schleicher, Maxwell A. Hockstein, Adriano R. Tonelli, Abhijit Duggal, Matthew T. Siuba

**Affiliations:** 1 Department of Internal Medicine, Community Care Institute, Cleveland Clinic, Cleveland, OH USA; 2 University Center of Volta Redonda, Rio de Janeiro, Brazil; 3 The Cleveland Clinic Floyd D. Loop Alumni Library, Cleveland Clinic, Cleveland, OH USA; 4 Departments of Emergency Medicine and Critical Care, MedStar Washington Hospital Center, Washington, DC USA; 5 Department of Critical Care Medicine, Respiratory Institute, Cleveland Clinic, Cleveland, OH USA; 6 Department of Pulmonary Medicine, Respiratory Institute, Cleveland Clinic, Cleveland, OH USA

**Keywords:** ARDS, Right ventricular dysfunction, Scoping review, Treatment

## Abstract

**Objective:**

To summarize knowledge and identify gaps in evidence regarding treatment of right ventricular dysfunction (RVD) in acute respiratory distress syndrome (ARDS).

**Data sources:**

We conducted a comprehensive search of MEDLINE, Embase, CINAHL, Web of Science, and the Cochrane Central Register of Controlled Trials.

**Study selection:**

Studies were included if they reported effects of treatments on right ventricular function, whether or not the intent was to modify right ventricular function.

**Data extraction:**

Data extraction was performed independently and in duplicate by two authors. Data items included the study design, patient population, type of intervention, comparison group, and RV-specific outcomes.

**Data synthesis:**

Of 1,430 studies screened, 51 studies reporting on 1,526 patients were included. By frequency, the included studies examined the following interventions: ventilator settings (29.4%), inhaled medications (33.3%), extracorporeal life support (13.7%), intravenous or oral medications (13.7%), and prone positioning (9.8%). The majority of the studies were non-randomized experimental studies (53%), with the next most common being case reports (16%). Only 5.9% of studies were RCTs. In total, 27% of studies were conducted with the goal of modifying RV function.

**Conclusions:**

Given the prevalence of RVD in ARDS and its association with mortality, the dearth of research on this topic is concerning. This review highlights the need for prospective trials aimed at treating RV dysfunction in ARDS.

**Supplementary Information:**

The online version contains supplementary material available at 10.1186/s13054-023-04395-9.

## Introduction

Acute respiratory distress syndrome (ARDS) occurs in approximately 10% of patients admitted to intensive care units (ICUs), carrying a mortality rate of nearly 35% [1]. Determining an accurate incidence and clarifying the epidemiology of right ventricular dysfunction (RVD) incurred during ARDS has been historically complicated by the lack of a universal definition of RVD. RVD occurs in anywhere from 20 to 50% of ARDS cases [2] and is associated with a nearly 50% increase in mortality [3, 4].

The pathophysiology of RVD in ARDS is multifactorial and incompletely understood. ARDS-mediated interstitial pulmonary edema, hypoxemic and hypercapnic pulmonary vasoconstriction, thromboembolism [5], and maladaptive vascular remodeling contribute to increased pulmonary vascular tone and right ventricular (RV) afterload [6, 7]. The alveolar inflation pressures associated with positive pressure ventilation and the use of positive end expiratory pressure (PEEP) to recruit the ARDS lung may also contribute to this increase in RV afterload [8]. The cumulative effect of both disease-specific complications and consequences of therapeutic interventions may ultimately lead to RV dilation and dysfunction [9]. There are no consensus guidelines on the screening, diagnosis, or management of RVD in ARDS. Several management strategies have been suggested under the moniker of “RV protective ventilation.” These include, but are not limited to, the following: prone position ventilation, adjustment of ventilator settings to limit inflation pressures and reduce hypoxemia and hypercapnia, inhaled pulmonary vasodilators, and extracorporeal life support (ECLS).

While previous reviews have described the management of pulmonary hypertension and RVD in adult critical care [2, 10, 11], there are no systematic assessments of the quantity and quality of evidence of interventions directed to manage RVD in ARDS. Given the high prevalence and impact of this condition, we aimed to assess the existing treatment strategies in this population. We hypothesized that the literature consists of a limited number of heterogeneous studies and thus performed a scoping review to describe the existing literature.

## Methods

### Protocol and registration

The study protocol and search strategy are registered on Open Science Framework (OSF) at the following link (https://osf.io/9kuph). Ethics approval was not necessary for this work. Reporting of this scoping review follows the Preferred Reporting Items for Systematic reviews and Meta-Analyses extension for Scoping Reviews (PRISMA-ScR) guidelines, and the checklist is included in Additional file 1. A scoping review strategy was chosen based on a priori suspicion that there would be few randomized controlled trials or large observational studies to synthesize.

### Information sources, search strategy, and eligibility criteria

MEDLINE, Embase, CINAHL, Web of Science, and the Cochrane Central Register of Controlled Trials databases were queried on July 21, 2022. Specific search terms can be found in Additional file 2. In brief, articles describing both ARDS and any mention of right ventricular function or dysfunction were included in the search. Studies in the English language with patients 18 years and older were included from any year. Animal studies were excluded.

### Selection of sources of evidence

All study types were eligible for inclusion except for review articles. Conference abstracts were excluded due to high risk of bias. Studies were included if they reported changes in RV function (by any metric) before and after an intervention, whether it was intended to directly influence RV function or not.

### Data extraction process and data items

Covidence software (Covidence systematic review software, Veritas Health Innovation, Melbourne, Australia) was used for abstract and full-text screening. Abstract screening was completed by two authors (SG and GA) independently, in duplicate, and conflicts were resolved by a third author (MTS). Full-text screening was completed by MTS. Data extraction was performed independently and in duplicate by authors SG and MTS. Data items included the study design, patient population, type of intervention, comparison group (when available), outcome, and any additional information felt useful to describe the literature.

### Synthesis of results

Results were summarized by intervention type, including the following categories:

1. Changes to ventilator settings, such as PEEP, tidal volume (Vt), respiratory rate, or ventilator mode
2. Prone position ventilation (PP)
3. Inhaled pulmonary vasodilators
4. Intravenous or oral medications
5. ECLS.

Outcome summarization could not be reported uniformly due to heterogeneous use of technologies and parameters to assess RV function. Therefore, we reported the outcomes provided by the individual studies, which typically included transthoracic or transesophageal echocardiographic measurements of RV size and function and/or pulmonary artery catheterization (PAC) determinations. Given the heterogeneity of the literature, no formal synthesis or analyses were performed.

## Results

### Selection of sources of evidence

A total of 1430 studies were assessed for abstract review after removing duplicates. After exclusion of 1317 due to irrelevance, 113 full-text studies remained. In total, 62 studies were further excluded after a full-text review (Additional file 2: Fig. S1). We report the data on 1526 patients from the remaining 51 studies.

### Characteristics of sources of evidence

We identified 27 (53%) non-randomized experimental studies, 8 (16%) case reports, 5 (9.8%) prospective cohort studies, 4 (7.8%) retrospective cohort study, 3 (5.9%) case series, 3 (5.9%) randomized controlled trials (RCT), and 1 (2%) cross-sectional study. Fifteen studies (29.4%) evaluated ventilator settings, 17 (33.3%) studied inhaled medications, 7 (13.7%) each tested ECLS and IV/PO medications, and 5 (9.8%) tested prone position ventilation.

**Fig. 1:**
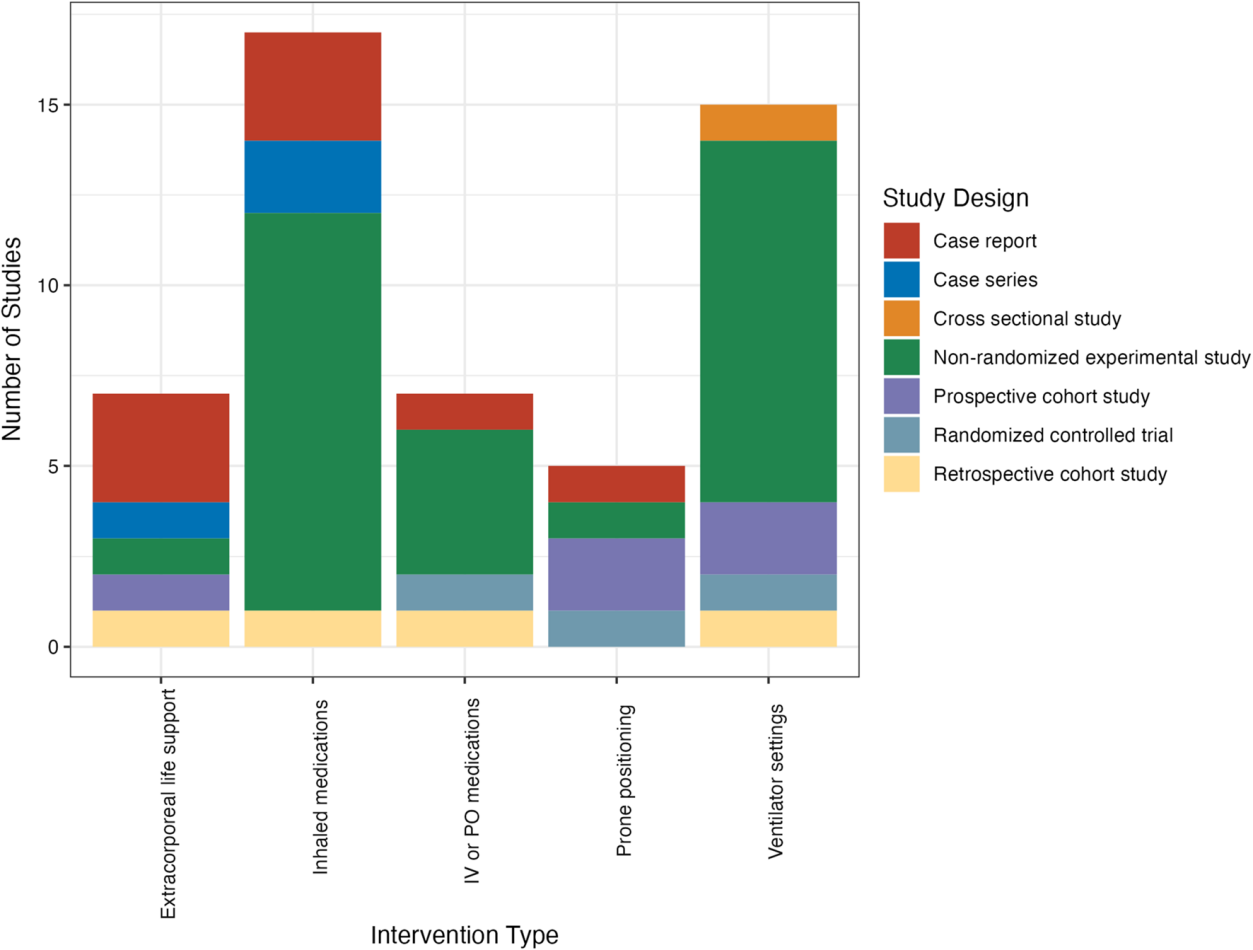
Interventions studied, by study design. IV = intravenous, PO = by mouth

**Fig. 2:**
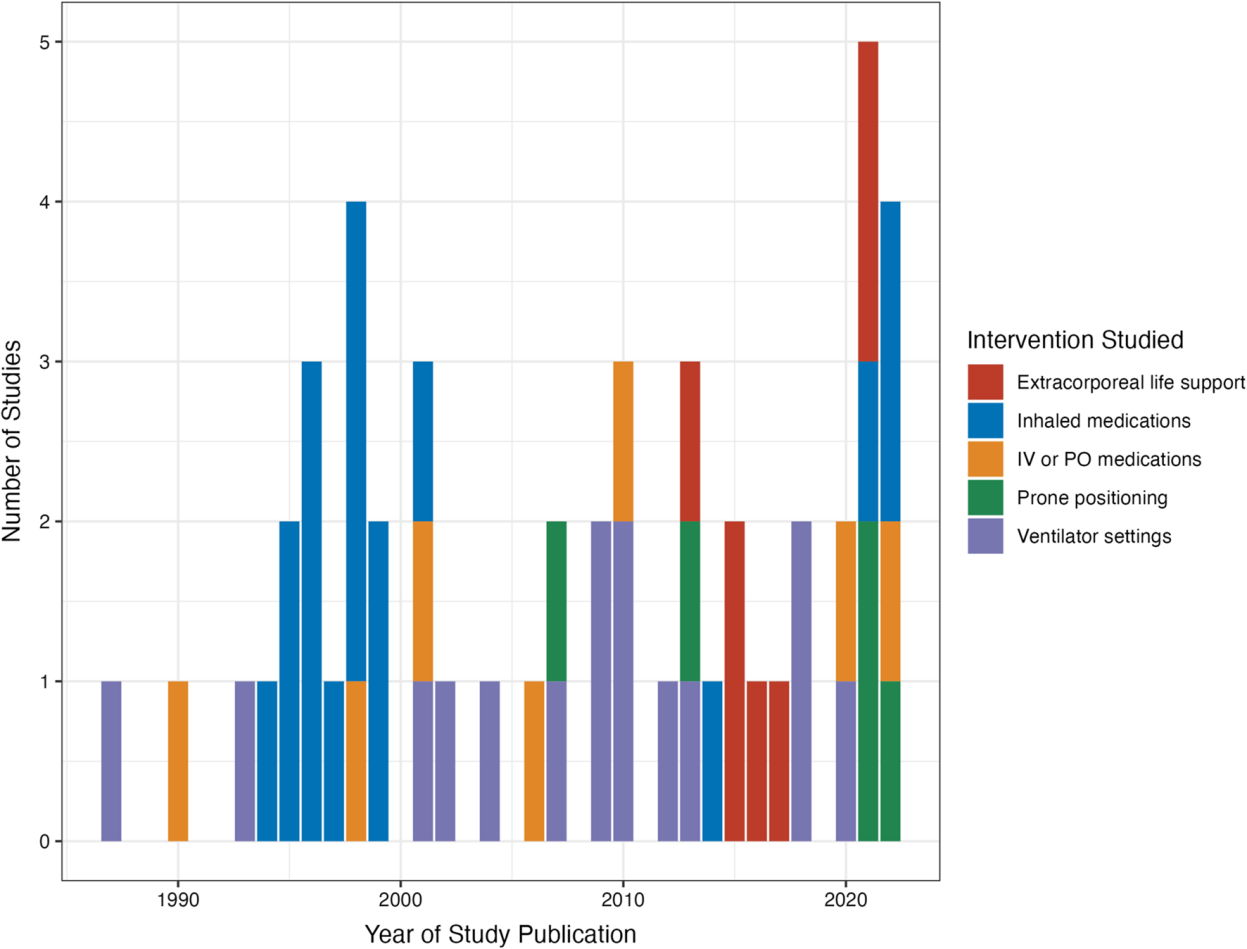
Interventions studied by year. IV = intravenous, PO = by mouth

The median number of patients enrolled in all studies was 13 (interquartile range [IQR] 8–20); by intervention: ECLS (*n* = 6, IQR 1–16), inhaled medications (*n* = 12, IQR 8–16), IV/PO medications (10, IQR 8–26), PP (18, IQR 9–42), and ventilator settings (*n* = 16, IQR 12–20) (Fig. 1 and Additional file 2: Table S1). Only 14 studies (27%) were conducted with the goal of treating RVD; 50% of these assessed the effect of inhaled medications, with 29% and 21% assessing IV/PO medications or ECLS, respectively. No studies were conducted with the goal of preventing right ventricular dysfunction. Interventions studied by year are detailed in Fig. 2. Study design by year is detailed in Additional file 2: Fig. S2. A total of six studies (11.8%) reported on patients with COVID-19.

RV function was assessed by various means, the most common of which was by PAC measurements (*n* = 31, 60.7%), followed by transthoracic echocardiography (TTE) (*n* = 16, 31.4%) and transesophageal echocardiography (TEE) (*n* = 11, 21.6%) (Additional file 2: Table S2). Other means of RV function assessment included cardiac magnetic resonance imaging, central venous catheter pressure measurements, invasive pressure–volume loop measurements, and transpulmonary thermodilution systems.

### Ventilator settings

Out of the 15 studies testing ventilator settings, 9 tested the effect of PEEP on RV function. Full details of the studies are listed in Additional file 2: Table S3. Four studies showed increasing PEEP led to RVD by observing unfavorable responses such as decreased stroke volume (SV), increased right ventricular end diastolic volume (RVEDV), decreased tricuspid annular plane systolic excursion (TAPSE), and decreased cardiac index (CI) [12–15]. One study showed no statistically significant impact of PEEP on CI [16]. Another compared PEEP set to best respiratory system compliance to PEEP set using the lower inflection point of P–V curve, demonstrating better RV function with best compliance PEEP compared to PEEP set using the lower inflection point [17]. One study examined recruitment maneuvers, including a sustained insufflation to 45 cmH2O for 40 s, and showed decreased CI and increased mean pulmonary artery pressure (mPAP) during the maneuver [18]. Two other studies evaluating the impact of recruitment maneuvers in this patient population found that although the RV Tei index, cardiac output (CO), and TAPSE were lower at time of the maneuver, they returned to baseline with decremental PEEP titration to best respiratory compliance [19, 20].

Three studies evaluated high-frequency oscillatory ventilation (HFOV), showing a reduction in CI, increased right ventricular end diastolic area (RVEDA): left ventricular end diastolic area (LVEDA), increased mPAP, and pulmonary vascular resistance (PVR) index during HFOV compared to standard ventilator settings [21–23]. Another investigation compared inverse ratio, pressure control ventilation to volume control ventilation, showing a significant increase in CI in inverse ratio ventilation compared to volume control with no significant change in mPAP [24].

Another study evaluated the impact of various plateau pressures on the incidence of acute cor pulmonale (ACP). It is found that the incidence of ACP as well as mortality was highest in patients with plateau pressures > 35 cmH_2_O. It is also noted that in patients with plateau pressures ranging from 18 to 26 cmH_2_O, the presence of ACP did not confer increased mortality compared to those without ACP [25]. One study attempted to measure the impact of respiratory rate on RVD, showing a higher respiratory rate resulted in a lower CI and pulmonary artery (PA) velocity time integral (VTI) [26]. This was due in part to higher alveolar dead space, as well as increase in intrinsic PEEP.

### Prone positioning

Out of the 5 studies testing prone positioning, 3 showed improvement in RV function by a reduction in RVEDA:LVEDA ratio and increased CI (Additional file 2: Table S4) [27–29]. In contrast, a case report showed a decrease in SV and RV EF, and increased mPAP as measured with invasive pressure–volume loops [30]. Another study demonstrated no significant impact of prone positioning on RV function as measured by TTE [31].

### Inhaled pulmonary vasodilators

Of the 17 studies evaluating inhaled pulmonary vasodilators, 15 tested inhaled nitric oxide (iNO), 3 tested nebulized prostacyclin analogues, and 2 tested nebulized alprostadil (Additional file 2: Table S5). A wide range of iNO doses were used, ranging from 5 to 40 parts per million (ppm). Eleven of the studies with iNO showed a reduction in mPAP and/or PVR/PVRi [32–42], 3 showed improvement in RV EF [33, 35, 38], and 4 showed improvement in CO [37, 41–43]. In contrast, 6 studies failed to find a statistically significant impact of iNO on CO [32–34, 39, 40, 44]. Two investigations reported reduced mortality in patients who responded to iNO (40% vs 67% in one study and 50% vs 65% in the other) [35, 41], and another noted return to baseline hemodynamic measurements postdiscontinuation of iNO [37].

Three studies evaluating nebulized prostacyclin demonstrated a reduction in mPAP. Two of those also showed a reduction in PVR. There was no change in CO/CI in any of the studies [36, 44, 45].

Two studies investigating nebulized alprostadil showed no significant change in CI as measured by PAC, although one showed a decreased in right ventricular end systolic and end diastolic volume index [38, 46].

### Intravenous and oral medications

Two studies evaluating enteral sildenafil found a decreased PVR with an increase in CO [47, 48]. Two studies tested IV almitrine; one demonstrated a decrease in mPAP and PVR, an increase in CI, and an improved RV global longitudinal strain at a dose of 4 μg/kg/min [43]. The other investigation found that almitrine increased mPAP, PVRi, and reduced RV ejection fraction (EF) without a significant change in CI at a dose of 16 μg/kg/min [49]. An investigation of IV epoprostenol showed a reduction in mPAP and PVRi, and an increase in RV EF (in patients with abnormal baseline values) and CI [50]. Another study explored the use of IV norepinephrine with iNO compared to iNO alone and found that there was a greater decrease in mPAP, PVR index and right ventricle stroke work (RVSWI) in patients receiving norepinephrine with iNO compared to iNO alone [51]. A single RCT in 35 patients demonstrated that levosimendan decreased mPAP, PVR index, and increased CI (from 3.8 L/min/m2 to 4.2) as well as RVEF [52]. See Additional file 2: Table S6.

### Extracorporeal therapies

Three out of the seven studies exploring the impact of ECLS evaluated veno-venous extracorporeal membranous oxygenation (VV-ECMO). These works have heterogeneous findings, with one showing decreased central venous pressures (CVP) and mPAP without a significant change in CO [53], while another case report showed decreased interventricular septal flattening immediately after VV ECMO cannulation, which normalized at time of ICU discharge [54]. Another investigation demonstrated that VV-ECMO combined with ultra-protective tidal volumes (driving pressures of 10 cm H2O) reduced PA systolic pressures; however, it did not result in significant differences in RV size and systolic dysfunction as measured by TTE [55]. In a case series, the addition of an intra-aortic balloon pump (IABP) to VV-ECMO resulted in increased PA pressures and reduced CVP [56]. A case report displayed a reversal of septal flattening and improved RV systolic function on conversion to VAV-ECMO from VV-ECMO [57].

Two studies examined the impact of extracorporeal CO2 removal (ECCO_2_R) on RV function. One found that ECCO_2_R decreased RVEDA, improved RVEDA:LVEDA ratio, and increased CO [58]. The second study investigated the impact of ECCO_2_R with ultra-protective TV (4 ml/kg of ideal body weight) and found no detectable change in CVP, right ventricular end diastolic diameter to left ventricular end diastolic diameter ratio, or PA systolic pressures; however, it did show improvement in TAPSE, see Additional file 2: Table S7 [59].

## Discussion

To our knowledge, this is the first scoping review of the literature on the effect of clinical interventions on RV function in ARDS. Despite over 50 years of ARDS research, we found only 51 citations meeting inclusion criteria, only 27% of which intended to directly influence RV function. The strength of evidence overall is low, given the low number of enrolled patients and a paucity of randomized controlled trials—only three in this study. The majority of more recent studies were case reports, case series, or otherwise observational in nature. The sole pharmacologic RCT on RVD in ARDS occurred in 2006 [52]. Overall, the literature on RVD in ARDS suffers from a lack of standardization in definitions, treatment targets, and patient-centered outcomes.

Significant heterogeneity in the included literature precludes meaningful pooling and systematic synthesis of these results. Many of the interventions tested were applied at different doses and durations with differing inclusion and exclusion criteria. A significant proportion of studies tested inhaled pulmonary vasodilators, which have been repeatedly shown to improve oxygenation, with uncertain effect on pulmonary artery pressures, and no clear mortality benefit [60, 61]. Over the past decade, extracorporeal life support modalities have been tested with increasing frequency, but there is still considerable heterogeneity in the configurations used as well as the quality of evidence.

Furthermore, methods of RV assessment and the definition of RVD differed across studies. The most common assessment was via pulmonary artery catheterization, which largely reflects the era in which most of these studies were completed. Currently, pulmonary artery catheters are used infrequently in patients with ARDS in many centers around the world. While transthoracic echocardiography has become increasingly available across intensive care units, the challenges of acquiring adequate images for RV assessment in patients with ARDS may limit its utility. Furthermore, approximately 30% of included studies were completed before the lung protective ventilation era, where higher tidal volumes (and thereby distending pressures) were used. This may have predisposed to higher rates of RVD [4], perhaps in ways which could modify responsiveness to any of the tested therapies compared to a contemporary cohort.

### Priorities

In order to enroll patients in high-quality prospective studies, consensus definitions of RVD and specifically, RVD in ARDS, are needed [62]. These metrics ideally should account for both functional and structural changes in the right ventricle, incorporating features such as systolic and diastolic function, size, morphology, as well as measures of right ventricular pulmonary artery coupling [63]. Reproducible definitions of RVD may be aided by improved cardiovascular phenotyping in ARDS [64, 65]. While the incidence of RVD in ARDS is estimated to be 20–50% [2], both the etiology and the natural history of RVD in ARDS are incompletely understood and could have time-varying characteristics.

Given the mortality risk associated with RVD in ARDS, this review highlights several priority areas for scientific inquiry. There is a clear need for randomized controlled trials of all the major categories of treatments detailed in this review. A “RV-protective ventilation” strategy has been described [7] and would be a low-cost intervention bundle with potentially high benefit. Patients at increased risk for RVD in ARDS (by established acute cor pulmonale risk scores [4]) could be selectively enrolled in trials, even as a preventative strategy. While patients with moderate to severe ARDS seem to benefit from a higher PEEP strategy [66, 67], optimal PEEP strategy may differ in patients at increased risk for RVD. While pulmonary vasodilator medications have not shown benefit in unselected ARDS patients, it may be worthwhile to conduct RCTs in patients with established RVD as a predictive enrichment strategy.

Although previous investigations examining the use of extracorporeal support in ARDS have yielded tepid results [68, 69], RVD is not typically an explicit therapeutic target for ECLS. However, RVD is associated with increased mortality in this group [70]. In addition to the included studies on ECLS, a retrospective analysis of 15 patients with COVID-19 ARDS demonstrated improvement in RV echo parameters after VV-ECMO cannulation (published after our systematic search). In patients who are already receiving ECLS, there are still opportunities for prospective physiologic studies to mitigate RVD, using adjunctive management such as pharmacologic therapies as well as different mechanical ventilation strategies. One recently published retrospective analysis demonstrated an improvement in RVD and hemodynamic parameters when PEEP was optimized to a slightly positive transpulmonary pressure using esophageal manometry [71]. Finally, there may be differences in right ventricular outcomes based on cannulation strategy [72, 73], which should be further investigated in prospective studies. Rescue strategies (including changes to cannulation strategy, among other interventions) for persistent RVD despite ECLS [74] deserve further study but may be challenging to perform systematically across a small number of heterogeneous patients and centers.

Secondly, there are several areas of ARDS management which were unexplored in this review of the literature. For instance, the specific role of tidal volume was not studied. This may be a priority area since it has been demonstrated that higher tidal volumes increase RV afterload even outside of ARDS [75]. The effect of tidal volume on mortality in ARDS may depend on respiratory system elastance [76], and it is unknown if any of this effect is due to impact on RV function. While some alternative modes of ventilation were studied, including inverse-ratio pressure control, no studies on airway pressure release ventilation were found. Finally, the effect of commonly used vasoactive agents which may have varying effects on pulmonary vascular resistance (such as vasopressin and phenylephrine) as well as inotropic agents (such as dobutamine and milrinone) was not specifically assessed.

### Limitations

This review has limitations. We systematically and exhaustively searched multiple databases, but relevant articles may have been missed if the broad search criteria did not capture them. Extraction of data from individual studies proved challenging given heterogeneous reporting, which limited our own reporting to a descriptive format.

## Conclusions

Given the prevalence of RV dysfunction in ARDS and its association with mortality, the dearth of high-quality research in this area is concerning. The existing literature is characterized by small sample sizes, inconsistent application of treatments across studies, and variable reporting of results. Prospective trials aimed at treating or preventing RV dysfunction should be a research priority for the ARDS scientific community.

## Supplementary Information

**Additional file 1.** PRISMA-ScR Checklist.

**Additional file 2.** Search Strategy, Supplemental Tables and Figures.

## Data Availability

All data related to this work are included in the main text or the supplementary materials. For pre-registered search strategy, please see https://osf.io/9kuph.

## Abbreviations

RVD: Right ventricular dysfunction
ARDS: Acute respiratory distress syndrome
RV: Right ventricle
PEEP: Positive end expiratory pressure
ECLS: Extracorporeal life support
PP: Prone position ventilation
SV: Stroke volume
RVEDV: Right ventricular end diastolic volume
TAPSE: Tricuspid annular plane systolic excursion
CI: Cardiac index
mPAP: Mean pulmonary artery pressure
CO: Cardiac output
HFOV: High-frequency oscillatory ventilation
RVEDA: Right ventricular end diastolic area
LVEDA: Left ventricular end diastolic area
ACP: Acute cor pulmonale
PA: Pulmonary artery
VTI: Velocity time integral
iNO: Inhaled nitric oxide
PVR: Pulmonary vascular resistance
PAC: Pulmonary artery catheter
VV-ECMO: Veno-venous extracorporeal membranous oxygenation
ICU: Intensive care unit
IABP: Intraaortic balloon pump
CVP: Central venous pressure
ECCO_2_R: Extracorporeal CO2 removal
TV: Tidal volume
RCT: Randomized controlled trial
TTE: Transthoracic echocardiography
TEE: Transesophageal echocardiography
EF: Ejection fraction
RVSWI: Right ventricular stroke work index
