# Supplementary material for: Right ventricle-specific therapies in acute respiratory distress syndrome: a scoping review
Source: Crit Care. 2023 Mar 12;27:104. doi: 10.1186/s13054-023-04395-9 (PMC10008150; doi:10.1186/s13054-023-04395-9)

**Online Supplement**

Registered Search Strategy

Located at <https://osf.io/9kuph>

Ovid MEDLINE® ALL <1946 to July 20, 2022>

1 Ventricular Dysfunction, Right/ or Ventricular Function, Right/

2 ((right ventric* or right heart) adj5 (failure or function or dysfunction)).mp.

3 1 or 2

4 Respiratory Distress Syndrome/ or (acute respiratory distress syndrome or adult respiratory distress syndrome or ARDS).mp.

5 3 and 4

6 limit 5 to english language = 305

Embase <1974 to 2022 July 20>

1 heart right ventricle failure/ or heart right ventricle function/

2 ((right ventric* or right heart) adj5 (failure or function or dysfunction)).mp.

3 1 or 2

4 adult respiratory distress syndrome/ or (acute respiratory distress syndrome or adult respiratory distress syndrome or ARDS).mp.

5 3 and 4

6 limit 5 to english language = 721

CINAHL

S1 (MH "Ventricular Dysfunction, Right") OR (MH "Ventricular Function, Right")

S2 TI ( (right ventric* or right heart) N5 (failure or function or dysfunction) ) OR AB ( (right ventric* or right heart) N5 (failure or function or dysfunction) )

S3 S1 OR S2

S4 (MH "Respiratory Distress Syndrome, Acute") OR TI ( acute respiratory distress syndrome OR adult respiratory distress syndrome OR ARDS ) OR AB ( acute respiratory distress syndrome OR adult respiratory distress syndrome OR ARDS )

S5 S3 and S4

S6 S5 limited to English = 114

Web of Science (SCI & CPCI and BCI)

#1 TS= (right ventricular dysfunction or right ventricular function )

#2 TS= ((right ventric* ) NEAR/5 (failure or function or dysfunction)) or ((right heart ) NEAR/5 (failure or function or dysfunction))

#3 #1 OR #2

#4 TS= (Respiratory Distress Syndrome or acute respiratory distress syndrome or adult respiratory distress syndrome or ARDS)

#5 #3 AND #4 AND refined to English language

SCI & CPCI = 670

BCI = 231

Cochrane Central Register of Controlled Trials

Issue 7 of 12, July 2022

#1 [mh ^"Ventricular Dysfunction, Right"] OR [mh ^"Ventricular Function, Right"]

#2 ((right ventric* or right heart) NEAR/5 (failure or function or dysfunction)):ti,ab,kw

#3 #1 OR #2

#4 [mh ^"Respiratory Distress Syndrome"] OR ("acute respiratory distress syndrome" or "adult respiratory distress syndrome" or ARDS):ti,ab,kw

#5 #3 AND #4 = 158 (all from Trials – there is no English language filter in Cochrane)

**Supplementary Tables and Figures**

| **eTable 1. Study Size and Design by Intervention** | | | | | | |
| --- | --- | --- | --- | --- | --- | --- |
| **Characteristic** | **Overall**,  N = 51 | **Extracorporeal life support**,  N = 7 | **Inhaled medications**,  N = 17 | **IV or PO medications**,  N = 7 | **Prone positioning**,  N = 5 | **Ventilator settings**,  N = 15 |
| **Patients enrolled**  *N = 1,526 total* | 13 (8, 20) | 6 (1, 16) | 12 (8, 16) | 10 (8, 26) | 18 (9, 42) | 16 (12, 20) |
| **Study Design** |  |  |  |  |  |  |
| Case report | 8 (16%) | 3 (43%) | 3 (18%) | 1 (14%) | 1 (20%) | 0 (0%) |
| Case series | 3 (5.9%) | 1 (14%) | 2 (12%) | 0 (0%) | 0 (0%) | 0 (0%) |
| Cross sectional study | 1 (2.0%) | 0 (0%) | 0 (0%) | 0 (0%) | 0 (0%) | 1 (6.7%) |
| Non-randomized experimental study | 27 (53%) | 1 (14%) | 11 (65%) | 4 (57%) | 1 (20%) | 10 (67%) |
| Prospective cohort study | 5 (9.8%) | 1 (14%) | 0 (0%) | 0 (0%) | 2 (40%) | 2 (13%) |
| Randomized controlled trial | 3 (5.9%) | 0 (0%) | 0 (0%) | 1 (14%) | 1 (20%) | 1 (6.7%) |
| Retrospective cohort study | 4 (7.8%) | 1 (14%) | 1 (5.9%) | 1 (14%) | 0 (0%) | 1 (6.7%) |
| *^1^* Median (IQR); n (%) | | | | | | |

**eTable 2. Techniques for Right Ventricular Outcome Assessment**

| **Technique** | **N = 51** |
| --- | --- |
| Pulmonary artery catheter pressure and/or cardiac output measurements | 21 (41%) |
| Pulmonary artery catheter pressure and/or cardiac output measurements; Central venous catheter pressure measurements | 1 (2.0%) |
| Pulmonary artery catheter pressure and/or cardiac output measurements; Cardiac MRI | 1 (2.0%) |
| Pulmonary artery catheter pressure and/or cardiac output measurements; pressure-volume loops | 1 (2.0%) |
| Transesophageal echocardiography | 5 (9.8%) |
| Transesophageal echocardiography and transpulmonary thermodilution | 1 (2.0%) |
| Transesophageal echocardiography; Pulmonary artery catheter pressure and/or cardiac output measurements | 4 (7.8%) |
| Transesophageal echocardiography; Pulmonary artery catheter pressure and/or cardiac output measurements; transpulmonary thermodilution | 1 (2.0%) |
| Transthoracic echocardiography | 12 (24%) |
| Transthoracic echocardiography; Central venous catheter pressure measurements | 2 (3.9%) |
| Transthoracic echocardiography; Pulmonary artery catheter pressure and/or cardiac output measurements | 2 (3.9%) |
| n (%) | |

**eTable 3: Ventilator Settings and Modes, and Prone Positioning**

| **Study/Year** | **Type** | **Population and n** | **Ventilator Settings** | **Intervention** | **Was modifying RV function**  **the goal of the intervention?** | **Comparison** | **RV assessment method** | **RV outcomes** | **Secondary outcomes** | **Notes** |
| --- | --- | --- | --- | --- | --- | --- | --- | --- | --- | --- |
| Martin 1987 | Non-randomized experimental study | Day 1 of IMV  n=13 | Vt 12 to 17 ml/kg  FiO_2_ 0.6 to 0.8 to maintain PaO_2_ > 60 mmHg | PEEP increases in 5 cm H_2_O increments from 0 to 15 (up to 25 in six patients)  45 minutes at each PEEP level before measurement | No | PEEP of 0 | PAC | Reduced SV, BP, and RVEDV at PEEP 15 compared to PEEP 0  No detected change in RVEF and HR  PEEP 20-25 led to further decrease in RVEDV and SV | Volume infusion at PEEP 15 increased RVEDV and SV close to baseline values |  |
| Poelaert 1993 | Non-randomized experimental study | PaO_2_ of 70 mmHg despite PEEP of 10 cm H_2_O and more than 0.6 FiO_2_  n=12 | Attempted to keep Vt and PaCO_2_ constant after each ventilator adjustment | Pressure controlled inverse ratio ventilation: I:E ratio initially 1:1 -> 2:1 -> 4:1  30 minutes at each I:E ratio before measurement | No | Volume control ventilation | TEE and PAC | Cardiac index significantly increased ( 4.73±1.71 to 5.56±1.66 L/min/m^2^)  No detectable change in PA VTI, RV FAC  Increase in RVEDA at 4:1 compared to VC (117±33 vs 79±46) | No significant change in RAP, mPAP, PAWP  No significant change in calculated DO_2_ or VO_2_ |  |
| Schmitt 2001 | Non-randomized experimental study | P:F < 150, within 1-2 days of respiratory support  n=16 | Vt 8 ml/kg, I:E 1:2, average FiO_2_ 0.75  VC with constant flow | Highest PEEP that yielded the best respiratory system compliance | No | PEEP set to lower inflection point of P-V curve plus 2 cm H_2_O | TEE | Lower end-expiratory PA VTI, VMax, and stroke index at all in inflection point PEEP group vs best compliance PEEP | 38% mortality in the cohort | Average difference in PEEP between groups was 6 ± 4 cm H_2_O (higher in the inflection point group) |
| Vieillard-Baron 2002 | Prospective cohort study | First day of mechanical ventilation  n=14 | Vt 8 ml/kg, I:E 1:2 | High RR = 30 | No | Low RR = 15 | TTE | CI decreased from 3.3±0.7 to 2.9±0.6 at high RR  PA VTI decreased from 12.9 ±2.3 to 11.6 ±2.5 | 14% mortality in the cohort  Increased isovolumetric contraction time at high RR  Increased IVC diameter at high RR | Significant increase in aPEEP in at high RR (0.3±0.2 vs 6.4±2.7) as well as alveolar dead space |
| David 2004 | Prospective cohort study | P:F <200 with plan for HFOV ventilation  n=9 | Vt < 8 ml/kg in pressure control ventilation with PEEP ≥15, Paw(mean) ≥22 over 4 hrs, I:E 1:2) | HFOV in the first 30 mins  Set at 5 cm H_2_O above prior Paw(mean) | No | Prior settings | TEE and PAC | CI decreased from 3.53 (2.83-7.15) l/min/m^2^ to 2.95 (2.44-4.74) after 30 mins  No detectable change in mPAP | RAP and PAWP increased at 30 minutes |  |
| Jardin 2007 | Retrospective cohort study | ARDS patients from 1980 to 2006  n=352 | Not specified | Effect of various plateau pressures (PPlat) (18-26, 27-35 and >35 cm H2O) on mortality and RV function | No | NA | TTE | Incidence of acute cor pulmonale was 20%, 39% and 42% in the 3 PPlat groups from lowest to highest.  In the 18-26 range of PPlat, presence of acute cor pulmonale did not confer increased mortality compared to those with normal TTE. | 60% mortality in highest PP group versus 46% mortality in lowest. |  |
| Mekontso Dessap 2009 | Non randomized experimental study | P:F < 150, within 2 days of ARDS diagnosis  n=11 | As described | PEEP 10-11 = with Vt 6 ml/kg, RR 30, Heat-and-moisture exchanger used  vs  PEEP 10-11 = with Vt 6 ml/kg, RR 15, Heated humidified circuit used | No | PEEP 5 with 8 ml/kg Vt  Heat-and-moisture exchanger used | TEE | RV stroke index lower in both high PEEP strategies  LP: 22 (20-32), vs 17(10-26) with RR 30, 16 (11-27): with RR 15,  CI lower in both high PEEP strategies 1.87 (1.16–2.98) and 1.89 (1.38–3.35) for RR 30 and 15, respectively, vs LP strategy 2.6(1.53-3.5) |  |  |
| Gernoth 2009 | Non randomized experimental study | ARDS (lung injury score ≥2.5), within 96hrs of MV  n=12 | Pressure control with Vt between 5-8 ml/kg, I:E 1:1, RR to keep arterial pH >7.20 | Recruitment maneuver (starting at end-inspiratory pressure of 40 cm H_2_O for 2 minutes, max of 50 for 2 minutes), followed by decremental PEEP titration to best compliance +2 cm H_2_O | No | Baseline ventilator settings | TPTD, TEE | RV Tei index > 0.4 in 6/12 patients at baseline.  During RM, RV Tei index (0.39 ± 0.11 at baseline vs 0.42 ± 0.1 at RM), but improved at “best” PEEP (0.35 ± 0.11)  RVEDA increased during the RM (13.6 ± 3 at baseline vs 16.1 ± 4 cm2 at RM) but returned to baseline values at “best” PEEP.  CI and SV did not significantly change | LVEDA (17.3 ± 7 at T0 vs 13.5 ± 5 cm2 at T20/30) significantly decreased during maneuver  At “best” PEEP, LVEDA returned to baseline values. |  |
| Fougeres 2010 | Non randomized experimental study | ARDS with P:F 108 ± 40  n=21 | Volume control ventilation, constant flow, 6 ml/kg Vt at PEEP 5±1 | PEEP increased to obtain a plateau pressure of 30 cm H_2_O (average 13±4cm)  At higher PEEP, passive leg raising (PLR) was performed | No | Baseline values (low PEEP) | PAC, TEE | RVEDA/LVEDA significantly increased from 0.66±0.20 to 0.72 ±0.20, returned to baseline with PLR  CI at low PEEP 4.1 ± 1.4 vs  3.6 ± 1.2 at high PEEP (p < 0.05), vs  4.0±1.4 at high with PLR  PVR  310 ± 200 dynes sec cm^-5^ m^2^ at low PEEP,  385±188 at higher PEEP,  321±205 with PLR | No detectable change in mPAP |  |
| Iannuzzi 2010 | Randomized control trial | ARDS due to bacterial PNA, within 48 hr of ICU admission  n=40 | 6ml/kg Vt, PEEP 14, FiO_2_ 0.70 | Sustained inflation (SI): 45 cm H_2_O for 40 seconds | No | Pressure control (PC) ventilation: 45 cm H_2_O for 2 minutes with I:E 1:2, PEEP 16, RR 8 | PAC, TTE | mPAP increased with SI (40.4±7.8 to 49.6±2.8) and decreased with PC (40.2±5.7 to 30.2±3.5)  CI increased with PC (3.2±0.5 to 3.4±0.83) compared to SI (3±0.9 to 2.0±0.8  PVRi increased with SI (460.5±64.0 to 848.6±12.4) compared to  PC (505.0±20.1 to 247.0±15.2), non-significant | PaO2/FiO2 ratio increased after PC compared to SI  Left Ventricular end-diastolic and end-systolic areas decreased during both RMs, but the decrease was greater after SI than after PC.  Eccentricity index increased from baseline after the SI RM |  |
| Guervilly 2012 | Non randomized experimental study | P:F <150 at PEEP ≥8 within 48 hr of ARDS onset  n=16 | Baseline: VC with 6 ml/kg Vt, adjusted to plateau pressure < 30 cm H_2_O  PEEP set per ARDSnet | HFOV: three 1 hr periods with increasing mean airway pressure above the mean airway pressure in VC (+5, +10, +15) | No | Baseline ventilator settings | TEE, PAC | Results presented as: VC pre -> HFOV +5 -> HFOV +10 -> HFOV +15 -> VC post  RVEDA/LVEDA ratio: 0.64 (0.46-0.92) -> 0.6 (0.52-0.87) -> 0.91 (0.57 - 1.15) -> 0.90 (0.73-1.55) -> 0.64 (0.49-0.85)  RAP: 11 +/- 4 -> 12 +/- 4 -> 13+/-5 -> 15+/-7 -> 12+/-5  mPAP: 28 +/-7 -> 29+/-7 -> 33+/-9 -> 36+/-9 -> 27 +/-7  CI: 3.2 (2.6-4.1) -> 3.2 (2.6-4.1) -> 3.1 (2.2-3.7) -> 2.7 (2-3.6) -> 3.1 (2.4-3.5)  PVRi: 327 (240-412) -> 390 (281-554) -> 438 (267-600) -> 489 (311-838) -> 313 (274-514) | No detectable change in P:F ratio on HFOV |  |
| Fares 2013 | Cross sectional study | ARDS < 48 hr  n=367 | ARDSNet ventilation strategy | Various PEEP levels | No | NA | PAC | No detectable effect of PEEP on CI  PEEP ≤5  CI 4.2±1.1 L/min/m^2^  PEEP 8 - 10  CI 4.2±1.3  PEEP 12 - 15  CI 4.3±1.2  PEEP ≥16  CI 4.6±1.5 | No detectable difference in CVP, PAWP across PEEP groups | Secondary analysis of the FACTT trial |
| Mentzelopoulos 2018 | Non randomized experimental study | ARDS with P:F < 150, FiO_2_ 0.5 - 0.9 on PEEP 10-17, at least 24 hours of ventilation  n=17 |  | 4 Hz HFOV + 4 Hz HFOV with tracheal gas insufflation (TGI) | No | 7 Hz HFOV | TEE | RVEDA/LVEDA - higher during 7Hz compared to 4 Hz and 4Hz + TGI  Results presented as 4 Hz -> 4 Hz + TGI -> 7 Hz  TAPSE: 1.88 ± 0.47 -> 2.04 ± 0.55 -> 1.66 ± 0.48  Eccentricity index: 1.26 ± 0.1 -> 1.17 ± 0.13 -> 1.42 ± 0.17  CVP: 1.8 ± 0.5 -> 1.8 ± 0.5 -> 1.9 ± 0.5  CI (L/min/m2): 3.35 ± 0.29 -> 3.43 ± 0.36 -> 3.11 ± 0.4 | Oxygenation and shunt fraction improved during HFO |  |
| Mercado 2018 | Non randomized experimental study | Moderate to severe ARDS within 72 hr after diagnosis  n=20 |  | Stepwise recruitment maneuver (RM) from 25 cm H_2_O to max 40  RM followed by stepwise decrement in PEEP until optimal compliance reached (PEEP 14±5) | No | Baseline | TTE | Results presented as: baseline PEEP -> PEEP 40 -> and optimal PEEP  RVEDA/LVEDA  0.60 ± 0.12 -> 0.86 ± 0.14  -> 0.59 ± 0.13,  TAPSE:  1.93 cm ± 0.6 -> 1.51 ± 0.6 -> 1.9 ± 0.5  CO:  5.8 ± 2 ->  4.6 ± 2.3 -> 5.9 ± 2, | Non-responders (change in P:F ratio < 50%) had poorer hemodynamic tolerance to RM  LV and RV longitudinal strain were both impaired at RM and best PEEP |  |
| Zhao 2020 | Non randomized experimental study | 12 with ARDS,  6 with respiratory failure due to anticholinergic poisoning  n=18 |  | PEEP ≤ 5 cm H_2_O  vs 5 to ≤ 10  vs 10-15 | No | Lowest PEEP level (≤5) | TTE | TAPSE decreased from baseline to PEEP 10-15, 17.2 ± 2.5 cm vs. 11.1 ± 1.6  PVR increased from 2.4 ± 0.2 WU at baseline to 3.7 ± 0.3 at PEEP 10-15 | IVC diameter and CVP increased with increasing PEEP levels | Study described as “retrospective” in abstract but appears there was prospective enrollment |

**eTable 4: Prone Positioning**

| **Study/Year** | **Type** | **Population and n** | **Ventilator Settings** | **Was modifying RV function**  **the goal of the intervention?** | **Comparison** | **RV assessment method** | **RV outcomes** | **Secondary outcomes** | **Notes** |
| --- | --- | --- | --- | --- | --- | --- | --- | --- | --- |
| Vieillard-Baron 2007 | Prospective cohort study | Severe ARDS  50% of patients with acute cor pulmonale  n=42 | 8ml/kg Vt | No | N/A | TEE before and 18 hours after PP | Cor pulmonale group:  RVEDA:LVEDA decreased from 0.91 to 0.61  Decreased tricuspid regurgitation  Increased CI (2.9 to 3.4 L/min/m2)  No significant hemodynamic changes in the group without cor pulmonale | 19% hospital mortality |  |
| Jozwiak 2013 | Non-randomized experimental study | ARDS monitored with PAC and TPTD  n=18 | PEEP was titrated to obtain a plateau pressure of 28–30 cm H_2_O | No | N/A | PAC  TTE  TPTD for cardiac output | Decreased  PVRi  Reduced RVEDA/LVEDA  Increased RAP and PAOP  No sig change in mPAP | 56% and 44% ICU mortality in ‘no change in CO’ and ‘increase in CO’ cohorts, respectively  Increased intra-abdominal pressure | PEEP was adjusted in PP to keep the plateau pressure constant |
| Temperikidis 2022 | Prospective cohort study | COVID-19 ARDS  n=9 | Not mentioned | No | N/A | TTE | No significant impact of one PP session on RV free wall strain (whether it was abnormal or not to start) | 100% mortality in abnormal RV free wall strain group (n = 4), 20% (n = 1) in normal group | No patients with acute cor pulmonale included |
| Kremer 2021 | Case report | COVID-19 ARDS  n=1 | PEEP 10, driving pressure 14 | No | N/A | Invasive pressure-volume loop catheter  PAC | Increased end-systolic pressure (37 to 47 mmHg), end-diastolc pressure (16 to 29 mmHg), end-systolic volume (92 to 118 ml), end-diastolic volume (179 to 217 ml).  Decreased stroke volume (98 to 88 ml)  Decreased RVEF (58% to 44%)  mPAP increased from 31 to 39mmHg, CVP from 18 to 22 mmHg |  | No mention of cardiac output |
| Lu 2021 | Randomized controlled trial | Moderate to severe ARDS  n=80 | 6-8 ml/kg Vt  PEEP determined by ARDSnet table | No | Usual care | PAC 72 hours after intervention | mPAP, PVRi, RVEDVi more significantly reduced in PP group  RVEF increased more in PP group | 12.5% 28-day mortality in PP group compared to 22.5% in usual care group (non-significant) | Hemodynamic data only shown in figures and not otherwise quantified |

**eTable 5: Inhaled Medications**

| **Study/Year** | **Type** | **Population and n** | **Ventilator Settings** | **Intervention** | **Was modifying RV function**  **the goal of the intervention?** | **Comparison** | **RV assessment method** | **RV outcomes** | **Secondary outcomes** | **Notes** |
| --- | --- | --- | --- | --- | --- | --- | --- | --- | --- | --- |
| Bigatello 1994 | Non randomized experimental study | ARDS and pulmonary HTN (mPAP ≥25)  n=13 | Not specified | iNO 0-40 ppm  7/13 patients with prolonged iNO (2-27 days) | No | NA | PAC | mPAP decreased from 34±7 to 30±7 in short term (20ppm) and 38±7 to 31±6 in prolonged group (2-20 ppm)  PVR decreased from 228±152 dyn.s.cm-5.m -2 to 190±108 in short term group and from 241±68 to 181±50 in prolonged group  No detectable change in CO or CVP | 67% mortality in study  6/7 patients recieving prolonged NO died |  |
| Rossaint 1995 | Non randomized experimental study | Post-surgical ARDS, severe by Murray Score  n=10  n=6 also receiving VV-ECMO | Pressure control ventilation, Vt not reported  10-15 PEEP | iNO at 18 ppm followed by 36 ppm  Before or after concurrent IV epoprostenol (4 ng/kg) | yes | NA | PAC | 18 ppm NO:  mPAP reduced from 33 ±2 to 28± 1 mmHg, increased RVEF from 28±2 to 32±2  PVRi decreased from 151±14 to 132±20 dyn.s.cm-5.m -2.  Unchanged CO.  No change from above when NO was increased to 36ppm.  Intravenous PGI2: decreased PAP from 34+2 to 30+2mmHg and increased RVEF from 29±2 to 32±2, CI increased from 4.0±0.5 to 4.5±0.5 L/min/m^2^, PVRi decreased from 155±14 to 120±15 dyn.s.cm-5.m -2. | No detectable change in CVP |  |
| Fierobe 1995 | Non randomized experimental study | Severe ARDS by Murray Score, mPAP >30 mmHg  n=13 | Not specified | iNO at 5 ppm | yes | None | PAC | mPAP decreased by 4.8±4 mmHg  PVR decreased by 31±47 dyn-s/cm5  CO not detectably changed | ICU mortality 31% |  |
| Krafft 1996 | Non randomized experimental study | ARDS with P:F < 200, due to sepsis, requiring inotropic and vasopressor support  n=25 | Pressure control ventilation, PEEP 10-14, 4-8 ml/kg Vt | iNO at 18 ppm followed by 36 ppm | No | NA | PAC | 24% had decrease of mPAP ≥15% (responders)  PVR lower in responders than non-responders 258±73 vs 377±163 dyne·s·cm-5  RVEF increased to 40% in responders (from 36.9±6.6), EF decreased in non-responders | Overall mortality 56%, (40% responders, 67% non-responders) |  |
| Walmrath 1996 | Non randomized experimental study | ARDS (mean lung injury score 2.75±0.05)  n=16 | Not specified | Randomized to receive either iNO (Dose range 2 to 40ppm, mean dose 17.8±2.7ppm) first then aerosolized PGI_2_ (Dose range 1.5 to 34 ng/kg/min, mean dose 7.5±2.5 ng/kg/min) or vice versa | No | NA | PAC | iNO did not result in a statistically significant change mPAP or PVR or CO.  PGI_2_ reduced mPAP from 35±2.2 to 31.9±1.7.  PGI_2_ reduced PVR from 228±27.5 to 182±17 dyne·s·cm-5  PGI_2_: no significant change in CO |  | Dose titration performed to find the lowest effective dose for maximum effect on arterial oxygenation. |
| Zwissler 1996 | Non randomized experimental study | ARDS with P/F <155 despite optimal ventilator settings  n=8 | PCV, peak pressures up to 40, PEEP ≤10 | Aerosolized PGI_2_ administered at 3 escalating doses: 1, 10, and25 ng/kg/min followed by iNO administer at 3 escalating doses: 1, 4, and 8ppm  and | No | NA | PAC | PGI_2_ resulted in a significant decrease in mPAP at all three doses.  PVR was unchanged at 1ng/kg/min but reduced by 20% with the 10ng/kg/min dose without further decrease at the 25ng/kg/min dose.  No change in CI.  iNO decreased PAP at 4 and 8ppm, no change noted at 1ppm. no decrease in PVR. |  |  |
| Benzing 1997 | Case report | ARDS with acute cor pulmonale  P:F 55  n=1 | Peep=14, Pplat 34 | iNO at 20 ppm | yes | NA | TTE  PAC | mPAP reduced from 37 to 35 mmHg  CO increased from 5.2 l/min to 7.5 at 20ppm  PVR reduced from 322 dyne·s·cm-5 to 203  CVP reduced from 29 mmHg to 27  SV increased from 45 ml to 66  TTE from day 12 to day 14 (Post NO) revealed a reduced RV dimension, normal IV septum (previously paradoxical motion)  Most parameters returned back to previous value once iNO was discontinued |  |  |
| Putensen 1998 | Non randomized experimental study | ARDS  n=10 |  | Random order receipt:  iNO, inhaled prostaglandin E1 (PGE1), infusion of PGE1, no therapy | No | Control (self) | PAC | mPAP and PVR decreased and RVEF increased with all treatments  Aerosolized PGE1 and NO inhalation were associated with a decrease in RVEDVI and RVESVI at a constant CI.  Cardiac index was highest during intravenous PGE1 |  |  |
| Gallart 1998 | Non randomized experimental study | ARDS with P:F < 150 at FiO_2_ 1 without PEEP  n=48 | PEEP 10,  Vt titrated to PaCO_2_ 40-50 Ppeak < 35 | iNO 5ppm for 15 mins, then IV almitrine 4ug/kg/min for 1hr, then both medications combined | No | NA | PAC | mPAP decreased from 24±1 mmHg at baseline to 21.3 ±1 with iNO, increased with almitrine (26±1) decreased with combination (23±1)  PVRi decreased from baseline 404±25 dyne·s·cm-5 to 333±21 with iNO, increased with almitrine to 443± 30 and decreased with combination 353±19  No detectable change in CI | 52% mortality in the cohort |  |
| Iotti 1998 | Non randomized experimental study | Severe ARDS with positive response to 10ppm iNO (≥25% increase in PaO_2_)  n=8 (19 tested for iNO response) | Average PEEP 10.5 cm H_2_O | iNO randomised to increasing vs decreasing doses (range: 0.5 - 100 ppm) | No | NA | PAC | mPAP, PVRi decreased  (only shown graphically)  CI not changed |  |  |
| Bhorade 1999 | Non randomized experimental study | Acute right heart syndrome (mPAP > 30 mmHg with RV dilation on echo without left heart dysfunction)  requiring mechanical ventilation  n=26, n=12 with ARDS | Not specified | Inhaled NO from 10 to 80ppm | yes | NA | PAC | Significant increase in CO (20%), SV (7%), and 23% decrease in PVR  mPAP decreased by 8% | Mortality in whole group 65%, responders 79%, non responders 50% |  |
| Kuhlen 1999 | Case report | ARDS  n=1 | Pressure controlled ventilation  PEEP 12 | iNO at 10ppm with IV prostacyclin | yes | NA | PAC | Reduction of MPAP from 57 mmHg to 48 with either iNO or IV prostacyclin individually; combination 45  CO increased from 4.2 L/min to 5.9 with IV PGI2 or combination  PVR reduced from baseline 914 dyne·s·cm-5 to 580 with iNO, 488 with combination |  |  |
| Domenighetti 2001 | Non randomized experimental study | Moderate to severe ARDS with P/F <150  N=15 | “lung protective ventilation” | Nebulized PGI_2_ in dose ranging from 2ng/kg/min to 40ng/kg/min | no | NA | PAC | mPAP decreased from 32±1 to 29±1  PVR decreased 1 hour after PGI_2_ inhalation from 177±18 to 153±18 dyne.sec/cm^5^ |  |  |
| Mehra 2014 | Case series | Severe ARDS with right heart failure (systolic PA pressure > 30; RV > LV diameter or non-collapsible IVC)  n=4 | “lung protective ventilation” | iNO 5 - 10 ppm | no | NA | TTE | systolic PA pressure reduced in all cases  reduction in vasopressor need in all cases |  | No summary statistics |
| Heuts 2021 | Case report | COVID ARDS on VV ECMO  n=1 | Pressure control, inspiratory pressure 25 cm H_2_), PEEP 10, Vt 2.2 ml/kg | iNO at 30ppm | yes | NA | TTE | RV diameter improved from dilated to normal 12hrs after iNO  TR improved from severe to absent  CO increased from 6 L/min to 7.5  (unclear how measured) | Recirculation also improved | No quantitative values mentioned; received inhaled prostacyclin before iNO |
| Bonizzoli 2022 | Case series | COVID ARDS refractory to lung protective ventilation and pronation  n=12 |  | iNO at 40ppm | yes | NA | TTE before, 12hrs and 24hrs after iNO initiation | No detectable change in TAPSE, RV dimension, function or RV/PA coupling (TAPSE/RVSP) | 75% mortality in group | At baseline, 1/3rd of the population had RV dilation and dysfunction |
| Seo 2022 | Retrospective cohort study | Any patients ≥18yrs who received d ≥1 dose of nebulized alprostadil  n=54  n=5 ARDS/hypoxemia |  | Nebulized alprostadil | No | NA | PAC (n=22) | No significant change in and CI |  |  |

**eTable 6: Intravenous or Oral Medications**

| **Study/Year** | **Type** | **Population and n** | **Ventilator Settings** | **Intervention** | **Was modifying RV function**  **the goal of the intervention?** | **Comparison** | **RV assessment method** | **RV outcomes** | **Secondary outcomes** | **Notes** |
| --- | --- | --- | --- | --- | --- | --- | --- | --- | --- | --- |
| Radermacher 1990 | Non-randomized experimental study | ARDS patients with pulmonary hypertension (mPAP >= 28 mmHg)  n=8 | Tidal volumes 14-17 ml/kg body weight  PEEP 8-22 | Intravenous epoprostenol (12.5 - 35.0 ng/kg/min) for 45 minutes | yes | N/A | PAC | mPAP reduced from 35.6 to 29.1 mmHg  Cardiac index increased from 4.2 to 5.81 L/min/m^2  PVRi decrease from 5.1 to 2.5 mmHg/min/min^2/L  Increased RVEF in patients with abnormal baseline value | Increased venous admixture, no detectable change in PaO2, but increase in oxygen delivery (657 to 894 ml/min/m^2)  37.5% mortality |  |
| Papazian 1998 | Non-randomized experimental study | ARDS patients receiving inhaled nitric oxide during a 6 hour study period  n=27  (n=15 receiving norepinephrine) | Not specified | Intravenous norepinephrine | No | inhaled nitric oxide without norepinephrine | PAC | mPAP decreased more in patients receiving norepinephrine  PVRi decreased more in patients receiving norepinephrine  RVSWI decreased more in norepinephrine group | 52% mortality (40% in norepinephrine group, 67% in control, NS)  No effect of norepinephrine on gas exchange detected |  |
| Michard 2001 | Non-randomized experimental study | ARDS patients with P:F < 150  n=9 | Not specified | Intravenous almitrine (16 mg/kg/min) for 30 minutes | yes | N/A | PAC | mPAP increased from 31 to 33  PVRi increased from 353 to 397  RVEF decreased from 36 to 34  No sig change in CI | No change in arterial oxygenation or intrapulmonary shunt |  |
| Morelli 2006 | Randomized controlled trial | ARDS patients  n=35 | Vt 6-8 ml/kg IBW,  PEEP set at 12 cmH2O | Intravenous levosimendan (0.2 mcg/kg/min) for 24 hours without loading dose | yes | Placebo | PAC  Cardiac MRI | mPAP decreased from 29±3 to 25±3  PVRi decreased from 290±77 to 214±50 dynes/s/cm^5/m^2  DPG decreased from 7±2 to 4±2  Cardiac index increased from 3.8±1.1 to 4.2±1 L/min/m^2  Cardiac MRI:  RVESV index decreased, RVEF increased | Mixed venous oxygen saturation increased (62 to 70%) | More fluid administered to intervention group (6.6 vs 4.7 L). Fluid given targeted to PCWP goal |
| Cornet 2010 | Non-randomized experimental study | ARDS patients with P:F < 200  n=10  excluded “bilateral pneumonia” | Not specified | 50 mg sildenafil (enteral administration)  6 hour monitoring after administration | No | N/A | PAC | 30 min values:  mPAP decreased from 25 to 22 mmHg  PAWP decreased from 16 to 13 mmHg (  Cardiac index 3.4 to 3.7 L/min/m^2  RVSWI decreased  PVRI declined from 215 to 170 | Decreased MAP from 81 to 65 mmHg (P = 0.005), SVRi from 1,688 to 1,400 dynes*s/cm^5  PaO2 decreased from 87 to 70 mmHg  Shunt fraction increased from 24% to 31%  No change in PaCO2 (data not shown) | No data tables presented for hemodynamic results |
| Huette 2020 | Case report | COVID ARDS, P:F 70  n=1 | LPV | almitrine infusion at 4ug/kg/min | No | NA | TEE, PAC | 12 hours after infusion:  mPAP decreased from 42 to 36  DPG decreased from 25 to 22  CI increased from 2.5 to 3  PVR decreased from 5.9 to 4.5  TEE: septal dyskinesia resolved, decreased RV dilation  RV GLS improved from -14.8 to -22.7%. | Improvement in P:F from 70 to 283 |  |
| McFayden 2022 | Retrospective cohort study | Moderate to severe COVID ARDS  Pulmonary hypertension and/or RV dysfunction on TTE at baseline: RV FAC 38% (29-45), PVAT 95 ms (78.3 - 115.8)  n=25;  10 on VV ECMO  n=15 sildenafil post 6 days of iNO | Not described | Sildenafil 12.5 mg three times daily, titrated up to 25 mg if tolerated | yes | NA | TTE | PVR decreased from 2.35 WU (1.89-3.05) to 2.02 (1.57-2.58)  No detectable change in TAPSE or RVSP  Cardiac output (n=13) increased from 5.73 to 7.13 L min | 36% 90 day mortality  The P:F ratio increased in non-ECMO patients  Increased norepi dose i n ECMO patients after initiation of sildenafil | 68% of patients had pulmonary embolism  23 patients continued sildenafil at discharge |

**eTable 7: Extracorporeal therapies**

| **Study/Year** | **Type** | **Population and n** | **Ventilator Settings** | **Intervention** | **Was modifying RV function**  **the goal of the intervention?** | **Comparison** | **RV assessment method** | **RV outcomes** | **Secondary outcomes** | **Notes** |
| --- | --- | --- | --- | --- | --- | --- | --- | --- | --- | --- |
| Pappalardo  2013 | Case series | Patients receiving VV-ECMO + refractory right heart failure  n=4 (6 total, 2 without ARDS) | <6ml/kg Vt  PEEP reduced from 12±2 to 10±0 | Intra-aortic balloon pump added to VV-ECMO | yes | N/A | PAC | CVP decrease from 14 mmHg +/- 5 to 10 +/-1 after 24 hours  PA pressures increased (not quantified) | 4/6 patients survived | Limited reporting of RV dysfunction parameters by echocardiography |
| Lee 2015 | Case report | Patient on VV-ECMO for H1N1 ARDS  n=1 | Not mentioned | Conversion to VAV-ECMO | yes | N/A | TTE (qualitative) | Reversal of septal flattening  Improved RV systolic function | Discharged from hospital |  |
| Reis Miranda 2015 | Prospective cohort | Patients defined as having “acute respiratory failure”  n=13 |  | VV-ECMO, first six hours | No | N/A | PAC | 15 min after initiation:  mPAP decreased from 40 +/- 3 mmHg to 32 +/- 3  CVP decreased from 15 +/- 2 to 12 +/- 2  No significant change in CO as measured by thermodilution | No change in vasopressor dose over the study period |  |
| Cherpanath 2016 | Case report | ARDS secondary to pneumonia  n=1 |  | ECCO2R | No | N/A | TTE | RVEDA decreased from 26.4 cm2 to 20.3  RVEDA:LVEDA from >1.0 to only >0.6  CO increase from 4.9 to 6.0 L/min (measurement method not specified) moved to RV outcome | Discharged from hospital  Decreased norepinephrine dose | Assessment of efficacy confounded by dramatic decreases in tidal volume, PEEP (25 to 5 cm H2O) |
| Mongodi 2017 | Case report | ARDS secondary to pneumonia  n=1 | < 6ml/kg Vt, PEEP 8 | VV-ECMO | No | N/A | TTE  (qualitative) | Decreased interventricular septal flattening immediately after cannulation; normalized at ICU discharge | Discharged from hospital | Concurrent receipt of iNO |
| Goursaud 2021 | Non-randomized experimental study | Moderate to severe ARDS (P:F between 80 and 150)  n=18 | Befpre: Vt 6 ml/kg, PEEP to achieve Pplat 28-30 | ECCO2R with ultraprotective tidal volumes (4 ml/kg IBW) | yes | N/A | TTE | TAPSE increased from 22.9 to 25.4 mm  No detectable change in estimated CVP, RV/LV ratio, PASP | PaCO2 and pH worsened during the lower tidal volume period  38.9% 28-day mortality | Patients with pre-existing severe PAH excluded  Patients with extra-pulmonary ARDS and those with poor echo windows were excluded |
| Pettenuzzo 2021 | Retrospective cohort study | Severe ARDS  n=18 | PEEP: before 10, after 10  Vt: before 5.2, after 2.0ml/kg PBW | VV-ECMO with ultra-protective Vt (driving pressure 10 cm H2O) | No | N/A | TTE | No significant differences in RV size and systolic dysfunction after cannulation  PASP decreased from 58 to 46 mmHg  Sweep gas flow inversely correlated with PASP (r = -0.68)  PaO2 correlated with RV FAC (r = 0.95), data only available in 6 patients | RV systolic dysfunction was present in 9/14 (64%) of patients with reported parameters | 85.4% of patients during the study period were excluded due to absence of before/after cannulation images |

**eFigure 1: Study flow diagram**

**
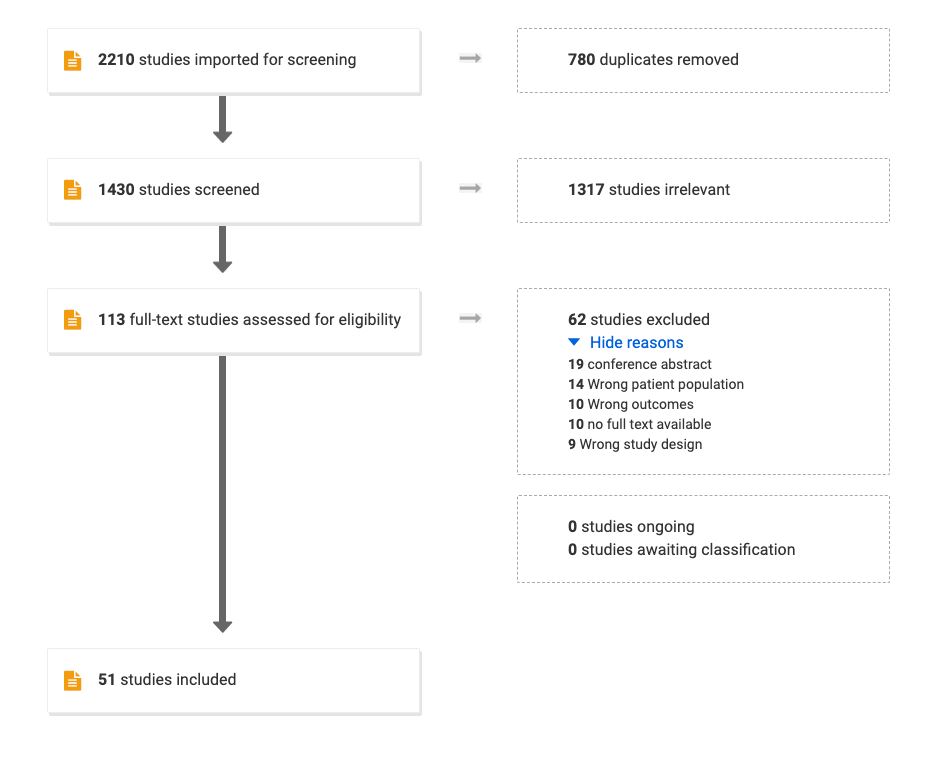
**

**eFigure 2. Study Design by Year**


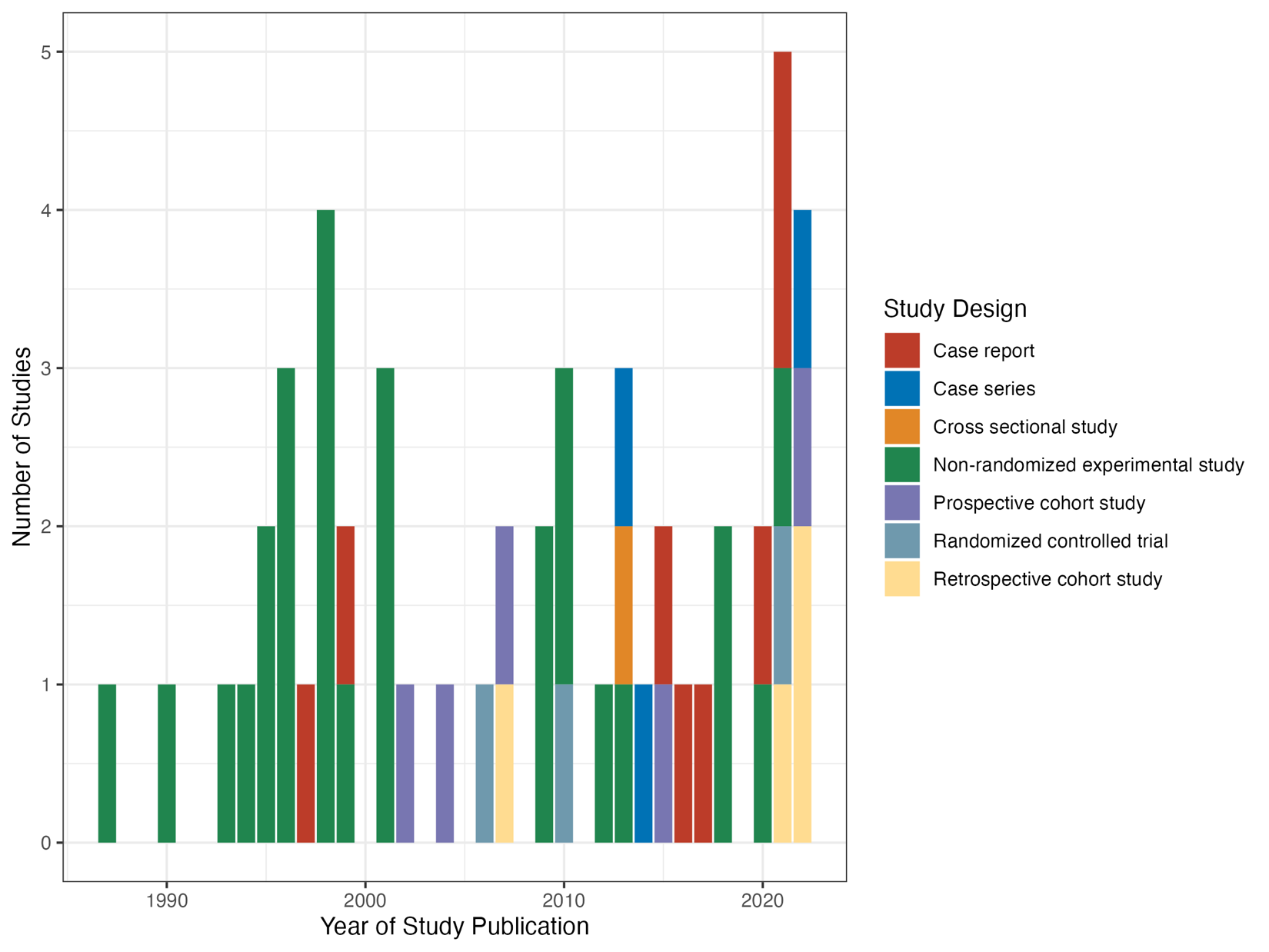

Supplement: Supplementary file 2 — Additional file 2. Search Strategy, Supplemental Tables and Figures. [file 13054_2023_4395_MOESM2_ESM.docx]
